# ﻿New species and new records of *Laccaria* (Agaricales, Basidiomycota) from Northern Thailand

**DOI:** 10.3897/mycokeys.107.127907

**Published:** 2024-08-07

**Authors:** Song-Ming Tang, Santhiti Vadthanarat, Bhavesh Raghoonundon, Zong-Long Luo, Xin-Yu Zhu, Feng-Ming Yu, Jun He, Shu-Hong Li

**Affiliations:** 1 College of Agriculture and Biological Science, Dali University, Dali 671003, China Biotechnology and Germplasm Resources Institute, Yunnan Academy of Agricultural Sciences Kunming China; 2 Biotechnology and Germplasm Resources Institute, Yunnan Academy of Agricultural Sciences, Kunming 650205, China Dali University Dali China; 3 Department of Biological Science, Faculty of Science, Ubon Ratchathani University, Ubon Ratchathani, 34190, Thailand Ubon Ratchathani University Ubon Ratchathani Thailand; 4 School of Science, Mae Fah Luang University, Chiang Rai 57100, Thailand Mae Fah Luang University Chiang Rai Thailand; 5 State Key Laboratory of Mycology, Institute of Microbiology, Chinese Academy of Sciences, Beijing, 100101, China Institute of Microbiology, Chinese Academy of Sciences Beijing China; 6 Key Laboratory for Plant Diversity and Biogeography of East Asia, Yunnan Key Laboratory of Fungal Diversity and Green Development, Kunming Institute of Botany, Chinese Academy of Sciences, Kunming 650201, Yunnan, China Kunming Institute of Botany, Chinese Academy of Sciences Kunming China; 7 School of Biotechnology and Bioengineering, West Yunnan University, Lincang, Yunan 677000, China West Yunnan University Lincang China

**Keywords:** 2 new taxa, Hydnangiaceae, phylogeny, taxonomy

## Abstract

Two new species *Laccariapseudoalba* and *L.subroseoalbescens* are described and illustrated, based on morphological characteristics and molecular phylogenetic analysis. Two new records, *Laccariaumbilicata* and *L.yunnanensis* from Thailand, are also reported. *Laccariasubroseoalbescens* is characterized by small basidiomata, stipe equal with an enlarged base, and nearly subclavate, pale pink to light orange. *Laccariapseudoalba* is characterized by pale orange to orange white pileus, has umbo when young on the pileus, and fistulose stipe of the pale to pastel red color. Phylogenetic analysis based on sequence data from rDNA internal transcribed spacer ITS1-5.8S-ITS2 rDNA (ITS), nuc 28S rDNA (28S), RNA polymerase II subunit 2 (rpb2), and translation elongation factor 1-α (tef1-α) are provided as further evidence. Molecular analysis confirms the phylogenetic positions of the two new species and two new records. The differences in characteristics of these two new species and closely related species are discussed herein.

## ﻿Introduction

The genus *Laccaria* Berk. & Broome, 1883 is a group of ecologically important ectomycorrhizal fungi that inhabit the soil (He et al. 2019). *Laccaria*, along with *Hydnangium* Wallr., *Maccagnia* Mattir. and *Podohydnangium* G.W. Beaton, Pegler & T.W.K. Young, belongs to the family Hydnangiaceae within the order Agaricales, phylum Basidiomycota.

Species of *Laccaria* are characterized by collybioid to omphaloid basidiomata; echinulate, acyanophilous, and inamyloid basidiospores; and a convex, plane, or umbilicate, hygrophanous pileus. Clamps are present in all parts of the basidiomata (Singer 1986; Mueller 1992; Latha and Raj 2019). Approximately 100 species of *Laccaria* have been described worldwide (according to Index Fungorum 2024), known to form symbiotic associations with plants of more than 20 genera (including *Abies*, *Castanea*, *Fagus*, *Pinus*, *Picea*, *Quercus*, *Larix*, *Lithocarpus*, and others) (Wilson et al. 2017). These associations benefit plant growth and facilitate effective nutrient acquisition (Smith and Read 2008). Therefore, studying *Laccaria* diversity is crucial for understanding terrestrial ecosystems and forest management.

*Laccaria* species are globally distributed and have been reported on every continent except Antarctica (Kropp and Mueller 1999). They have been found in association with both angiosperms and gymnosperms worldwide (Wilson et al. 2017) and form ectomycorrhizas (ECM) with many economically important plant species (Kropp and Mueller 1999). However, due to the morphological similarity among *Laccaria* species, defining species boundaries within the genus is challenging (Sheedy et al. 2013).

Since the establishment of *Laccaria* by Berk. and Broome (1883), many mycologists have contributed to its taxonomy (Orton 1960; McNabb 1972; Mueller 1984; Wang et al. 2004; Wilson et al. 2013, 2017; Popa et al. 2014; Popa et al. 2016; Luo et al. 2016; Cho et al. 2018; Li 2020). Historically, *Laccaria* was divided into *Russuliopsis* by J. Schröt, who only included species with a white spore print in *Laccaria* (Mueller and Vellinga 1986). To date, seven sectional names have been introduced within *Laccaria* (http://www.indexfungorum.org/Names/Names.asp; accessed date: 20 June 2024), leading to much controversy in its taxonomy.

The number of *Laccaria* species described from Asia has been increasing, with more studies focusing on Basidiomycetes. Since 2013, twenty-three new species of *Laccaria* have been described in Asia (Wilson et al. 2013; Popa et al. 2014; Luo et al. 2016; Popa et al. 2016; Cho et al. 2018; Li 2020; Cui et al. 2021; Zhang et al. 2023). Nevertheless, no *Laccaria* species were reported or described in Thailand during the same period.

Thailand is renowned as one of the world’s most important biodiversity hotspots with high fungal diversity (Hyde et al. 2018; Thongbai et al. 2018). During our recent investigation of *Laccaria* in Thailand, nine *Laccaria* specimens were collected. Based on morphological characteristics and phylogenetic analysis, two undescribed species and two new records have been identified. This paper provides detailed descriptions, illustrations, and phylogenetic analyses for these species.

## ﻿Materials and methods

### ﻿Morphological study

Specimens were collected from Chiang Mai Province, Thailand. They were photographed in the field, then separately wrapped in aluminium foil or kept in a plastic collection box. The fresh basidiomata were macro-morphologically described on the same day of collection. Colour codes were determined following Kornerup and Wanscher (1978). After being thoroughly dried at 50 °C (Hu et al. 2022) in a food drier, the specimens were stored in sealed plastic bags and deposited in Mae Fah Luang University Herbarium (**MFLU**) and Herbarium of Cryptogams Kunming Institute of Botany, Academia Sinica (KUN-HKAS). Dried materials were sectioned under a stereo microscope, transferred onto slides, and mounted in a 5% KOH solution. For microscopic characteristics, anatomical and cytological characteristics including basidia, basidiospores, and cystidia, were observed and photographed using a Nikon Eclipse 80i microscope at magnifications up to × 1000. For SEM studies, fragments of the lamellae of the dried material were taken, sputter coated with gold, and analysis with a Hitachi S520 (Hitachi Japan). The notation [x/y/z] specifies that measurements were made on x basidiospores measured from y basidiomata of z collections. At least 50 basidiospores and 20 basidia were measured from one basidioma. Basidiospores dimensions are given as (a–) b–c (–d). Where “a” and “d” refer to the minimum and maximum values of all measurements, respectively, b–c presents the range of 95% of the measured values, and *Q* is the length/width ratio of basidiospores, *Q*_m_ is the average *Q* of all basidiospores and is given as *Q*_m_ ± standard deviation.

### ﻿DNA extraction, PCR amplification, and sequencing

Genomic DNA was extracted from dried specimens using Ezup Column Fungi Genomic DNA extraction kit (Sangon China) following the manufacturer’s protocol. Primer pairs for PCR were respectively ITS1/ITS4 (White et al. 1990), LR5/LR0R (Vilgalys and Hester 1990), *rpb*2-5F/*rpb*2-7cR (Liu et al. 1999), and *tef*1-983F/*tef*1-2218R (Rehner and Buckley 2005). ITS, LSU, *rpb*2, and *tef*1 were amplified in 25 μL reactions containing 12.5 μL 2× Taq Plus Master Mix II (Vazyme Biotech Co., Ltd China), 9.5 μL ddH_2_O, 1 μL 10 μM of forward and reverse primers, 1 μL DNA. PCR conditions were carried out as follows in Table 1 using a C1000 thermal cycler (Bio-Rad China). The PCR amplicons were sent to Sangon Biotech (China) for Sanger sequencing. Sequence reads were assembled in SeqMan II (DNA STAR Inc.).

**Table 1.** PCR primers and cycling conditions used in the study.

Locus	Primers (Reference)	PCR conditions ^a^
ITS	ITS1, ITS4 (White et al. 1990)	94 °C: 30 s, 48 °C: 30 s, 72 °C: 1.00 min. (35 cycles)
LSU	LR0R, LR5 (Vilgalys and Hester 1990)	94 °C: 30 s, 48 °C: 30 s, 72 °C: 1.30 min. (35 cycles)
*rpb*2	f RPB2-5F, b RPB2-7cR (Liu et al. 1999)	95 °C: 30 s, 55 °C: 1 min, 72 °C: 1.30 min. (35 cycles)
*tef*1	983F, 2218R (Rehner and Buckley 2005)	95 °C: 30 s, 55 °C: 1 min, 72 °C: 1.30 min. (35 cycles)

^a^ The three steps given for each primer pair were repeated for 35 cycles, preceded by an initial denaturation step of 5 min at 94 °C, and followed by a final elongation step of 10 min at 72 °C and a final hold at 4 °C.

### ﻿Sequence alignment and phylogenetic analysis

The newly generated sequences were checked using BioEdit Sequence Alignment Editor version 7.0.4 and assembled using SeqMan (DNAstar, Madison, WI, USA). The sequences were then blasted using the Basic Local Alignment Search Tool (BLAST) against the GenBank database (Nilsson et al. 2006) to check the most closely related sequences. Reference sequences for a total of 103 specimens representing 55 species were retrieved (Table 2) and minimally adjusted by hand in BioEdit v.7.0.4 (Hall 2007) first, and then aligned using TrimAl (Salvador et al. 2009).

**Table 2.** *Laccaria* taxa and sample IDs with geographic location and GenBank ID numbers for ITS, LSU, *rpb*2, and *tef*1 sequences used in phylogenetic analysis. Sequences produced in this study are marked in bold. “*” following a species name indicates that the specimen is the holotype of that species.

Species name	Sample no.	Location	GenBank accession			
			ITS	LSU	*rpb*2	*tef*1
*Laccariaacanthospora*	HKAS45998	China	KU685719	KU685870	KU686069	–
*L.alba*	F1121461	China	JX504129	–	–	–
	ASIS18039	South Korea	MG519546	–	MG551620	MG551652
	TPML20120807-69	South Korea	MG519542	MG519583	MG551616	MG551649
*L.ambigua*	PDD89696*	New Zealand	KU685725	KU685876	KU686018	KU686132
*L.amethysteo-occidentalis*	AWW556	America	JX504107	JX504191	KU685919	–
	KGP40*	America	DQ822817	–	–	–
	DAVFP 28205	Canada	HQ650762	–	–	–
*L.amethystina*	GMM7041	Russia	KU685654	KU685797	KU685940	–
	GMM7621	France	JX504150	JX504224	KU686046	KU686152
*L.araneosa*	SFC2013091721*	South Korea	MG519549	MG519589	MG551622	MG551655
	TPML20120912-40	South Korea	MG519548	MG519588	MG551621	MG551654
*L.aurantia*	KUNF78557*	China	NR154113	–	–	–
	MBFB001109	Japan	JQ681209	–	–	–
*L.bicolor*	GMM7620	France	JX504149	JX504223	–	–
	HKAS44062	China	JX504159	JX504235	KU686068	–
	KA130253	South Korea	MG519524	MG519570	MG551599	MG551636
	GM7712	USA	KM067866	–	KU686012	–
*L.bullipellis*	AWW465*	China	JX504100	JX504184	KU685914	–
*L.canaliculata*	GMM7267	Australia	JX504137	JX504213	KU685960	KU686093
*L.fagacicola*	HKAS90435*	China	MW540806	–	–	–
	HKAS107731	China	MW540807	–	–	–
*L.fengkaiensis*	HKAS106739*	China	MN585657	MN621238	–	–
	HKAS106741	China	MN585658	–	–	–
*L.fulvogrisea*	KUN-F78556*	China	NR154114	–	–	–
	KUN-FB-101105	China	JQ681210	–	–	–
*L.guizhouensis*	HMAS352265*	China	OP244890	–	–	–
	HMAS352266	China	OP244891	–	–	–
*L.glabripes*	GMM7521	New Zealand	KU685708	KU685849	KU685991	KU686117
*L.himalayensis*	AWW463	China	JX504098	JX504182	KU685913	–
	AWW484*	China	JX504101	JX504185	KU685915	–
*L.japonica*	F64167*	Japan	KU962988	–	–	–
	SFC2012072212	South Korea	MG519518	MG519566	MG551595	MG551633
*L.longipes*	F1092175	America	KU685637	KU685780	–	–
*L.laccata*	GMM7615	France	JX504148	–	–	–
*L.macrocystidia*	GMM7616	France	KM067850	KU685863	KU686004	–
	GMM7612	France	KM067847	KU685861	KU686002	–
*miniata*	GDGM76043*	China	OR689440	OR785476		
*L.montana*	TWO591	America	DQ149865	–	–	–
	TWO319	America	DQ149862	–	–	–
*L.moshuijun*	HKAS 93732*	China	KU962989	–	–	–
	**HKAS 123302**	**China**	**ON557378**	**ON556493**	–	**ON598893**
	**HKAS 110653**	**China**	**ON557379**	**ON556494**	–	–
*L.murina*	ASIS216	South Korea	MG519553	–	–	–
	ASIS24249	South Korea	MG519552	MG519592	MG551625	MG551658
*L.nanlingensis*	GDGM 84954*	China	OR689442	OR785478	OR835199	OR826273
	GDGM 84949	China	OR689441	OR785477	OR835198	OR826274
*L.negrimarginata*	GMM7631	France	JX504152	JX504226	–	–
	BAP360*	China	JX504120	–	–	–
*L.neovinaceoavellanea*	GDGM52852*	China	OR689447	OR785479	–	–
	GDGM53063	China	OR689448	OR785480	–	–
	GDGM89621	China	OR689449	OR785481	–	–
*L.nobilis*	F1091206	America	KU685636	KU685779	–	–
*L.oblongospora*	ObiFr	France	GQ406466	–	–	–
*L.ochropurpurea*	PRL4777	America	KU685733	KU685883	KU686025	–
*L.ohiensis*	GMM7539	New Zealand	KU685712	KU685853	KU685994	KU686119
*L.prava*	A3394	Japan	JN942788	JN939770	JN993522	–
	ASIS19814	South Korea	MG519531	MG519575	MG551606	MG551642
	SFC2012091940*	South Korea	MG519525	–	MG551600	–
*L.prava*	HKAS106742*	China	MN585660	–	–	–
	HKAS106745	China	MN585661	–	–	–
*L.proxima*	F1081079	Argentina	KU685633	KU685777	KU685928	–
	GMM7584	Russia	KU685717	KU685858	KU685999	KU686120
***L.pseudoalba***	MFLU 22-0106*	**Thailand**	**ON557377**	**ON556492**	**ON598886**	–
	**HKAS 110664**	**Thailand**	**ON557376**	**ON556491**	**ON598887**	**ON598894**
*L.pseudomontana*	pse1625*	America	DQ149871	–	–	–
*L.pumila*	pum1252	America	DQ149864	–	–	–
*L.roseoalbescens*	LM5099*	Mexico	KJ874328	KJ874331	–	–
*L.rubroalba*	MS15	China	KX449358	–	–	–
	MS20	China	KX449357	–	–	–
*L.rufobrunnea*	GDGM82878*	China	OR689443	OR785482	OR835197	OR826272
	GDGM89627	China	OR689444	OR785483		
*L.salmonicolor*	GMM7596*	China	JX504143	JX504218	KU686045	KU686151
	GMM7602	China	JX504145	–	–	–
*L.squarrosa*	DM63*	Mexico	MF669958	MF669965	–	–
	SYC109	Panama	KP877340	–	–	–
***L.subroseoalbescens***	MFLU23-0339*	**Thailand**	**PP785397**	**PP789598**	–	–
	**MFLU23-0340**	**Thailand**	**PP785398**	**PP789599**	–	–
*L.tetraspora*	F1080957	Germany	KU685631	KU685775	–	–
*L.torosa*	SFC2015090217*	South Korea	MG519561	MG519598	MG551631	MG551664
	KA12-1306	South Korea	MG519562	–	–	–
*L.tortilis*	ASIS22273*	South Korea	MG519533	MG519576	MG551608	MG551644
	GMM7635	France	JX504155	KU685906	KU686053	KU686156
*L.trichodermophora*	F1111951	Costa Rica	KU685640	KU685784	KU686063	–
	GMM7733	America	JX504157	–	KU686013	–
*L.trullisata*	PRL7587	China	JX504170	JX504247	KU686047	KU686153
*L.umbilicata*	GDGM82883	China	OR689445	OR785485	OR835194	OR826270
	GDGM82911*	China	OR689446	OR785486	OR835192	OR826268
	**MFLU 22-0105**	**Thailand**	**ON557372**	**ON556490**	**ON598888**	**ON598896**
	**HKAS 110652**	**Thailand**	**ON557371**	**ON556489**	–	**ON598895**
*L.versiforma*	KNU2012100803	South Korea	MG519560	MG519597	MG551630	MG551663
	SFC20120926-01*	South Korea	MG519556	MG519594	MG551627	MG551660
*L.vinaceoavellanea*	A2986	Japan	JN942810	JN939738	JN993520	–
	A0559	Japan	JN942803	JN939756	JN993512	–
	SFC20150810-10	South Korea	MG519539	MG519580	MG551614	MG551646
*L.violaceonigra*	GMM7520	New Zealand	KU685707	KU685848	KU685990	–
*L.violaceotincta*	CAL1389*	India	MK141034	–	–	–
*L.yunnanensis*	KUNF78558*	China	NR154115	–	–	–
	**MFLU 22-0107**	**Thailand**	**ON557374**	**ON556488**	–	**ON598892**
	**HKAS 110636**	**Thailand**	**ON557373**	**ON556487**	**ON598889**	**ON598891**
	**HKAS 110638**	**Thailand**	**ON557375**	**ON556486**	**ON598890**	–
*Mythicomycescorneipes*	ES11.10.2.A	Germany	KC964108	–	–	–
	AFTOLID972	Germany	DQ404393	AY745707	DQ408110	DQ029197

Maximum likelihood (ML) analysis was performed separately for each locus and the concatenated dataset using RAxML-HPC2 v. 8.2.12 (Stamatakis 2014) as implemented on the CIPRES portal (Miller et al. 2010), with the GTR+G model for both genes and 1,000 rapid bootstrap (BS) replicates. For Bayesian Inference (BI), the best substitution model for each character set was determined with MrModeltest 2.2 (Nylander 2004) on CIPRES, using the Akaike information criterion. Bayesian analysis was performed using MrBayes ver. 3.2.7a (Ronquist et al. 2011) as implemented on CIPRES (Miller et al. 2010).

## ﻿Results

### ﻿Phylogenetic analyses

Thirty-three new sequences (11 of ITS, 11 of LSU, 5 of *rpb*2, and 6 of *tef*1) were generated for *Laccaria* species and deposited in GenBank (Table 2). The ITS dataset included 103 specimens representing 55 species, while the ITS-LSU-*rpb*2-*tef*1 dataset included 71 specimens representing 42 species. Two phylogenetic analyses were conducted: one for the 5.8S, ITS1+ITS2 dataset, and the other with concatenated matrix of 5.8S+LSU, ITS1+ITS2, *rpb*2 codon, *rpb*2 introns+*tef*1 introns and *tef*1 codons (Vaidya et al. 2011). The ITS final aligned matrix contained 687 positions (170 for 5.8S, 517 for ITS1+ITS2), while the concatenated matrix contained 3,509 positions (1,054 for 5.8S+LSU, 430 for ITS1+ITS2, 1,026 for *rpb*2 exons, 163 for *tef*1 introns+*rpb*2 introns, 836 for *tef*1 exons). Based on previous phylogenies (Wilson et al. 2013, 2017; Popa et al. 2014, 2016; Luo et al. 2016; Cho et al. 2018; Li 2020; Cui et al. 2021; Zhang et al. 2023), species of the *Mythicomycescorneipes* (Fr.) Redhead & A.H. Sm. were selected as the outgroup. In the 5.8S-ITS1-ITS2 dataset, the following models were selected by mrModelTest: SYM for 5.8S and GTR+I+G for ITS1+ITS2. In the ITS, LSU, *rpb*2, and *tef*1 datasets, the models selected by mrModelTest were: GTR+I+G for 5.8S+LSU and *tef*1 codon, GTR+G for ITS1+ITS2 and *rpb*2 codon, GTR+G for *rpb*2 introns+*tef*1 introns.

In MrBayes analysis, two runs of five chains each were run for 2,000,000 generations and sampled every 200 generations. Convergence was further evaluated by checking that the potential scale reduction factor (PSRF) statistic was close to 1 for all parameters. Moreover, the effective sample size (ESS) was much higher than 200 for all parameters. A clade was considered to be supported if showing a bootstrap support value (BS) ≥75% and/or a posterior probability (PP) ≥0.90. Trees were edited in FigTree version 1.4.0 and PowerPoint.

Fig. 2 presents the phylogeny from the combined datasets. Nine specimens collected in northern Thailand formed three monophyletic clades, here described as *L.pseudoalba*, *L.subroseoalbescens*, *L.umbilicata*, and *L.yunnanensis*, respectively. Each clade was well supported by both ML and BI in the concatenated trees (Fig. 2). In our phylogenetic analysis, the four species clustered as separate clades with high support. Thus, these species are formally described in this paper.

### ﻿Taxonomy

#### ﻿ Laccaria pseudoalba

Taxon classificationFungiAgaricalesHydnangiaceae

S.M Tang & S.H. Li sp. nov.

FDA1759E-DEDC-548F-B30C-DC2E62BFA05F

844144

Figs 3 , 4 , 5 , 12

##### Etymology.

The epithet “pseudoalba” refers to its similarity to *L.alba* in their small basidiomata and orange-white to pale orange pileus.

##### Holotype.

Thailand. Chiang Mai Province: Mae On district, Huay Keaw subdistrict, Pox village, 18°43'55.6"N, 99°17'50.1"E, elevation 789 m., 6 September 2020, S. M. Tang, 2020090608 (MFLU 22-0106).

##### Description.

Basidiomata small. Pileus 9–15 mm in diam., convex to applanate, hemispherical, applanate to plano-concave, pale orange (5A2–3, 6A2–3), orange-white (5A2–3, 6A2–3), when dry moisture loss of moisture or with age becoming whitish, clearly striate on the surface; umbo when young, becoming papilla to abrupt papilla with age; margin inflexed, sometime reflexed; context thin, 1–2 mm, pale orange (5A2–3), unchanging. Lamellae distant, arcuate, adnate with decurrent tooth, orange white (5A2–3, 6A2–3) when young, become pale orange with age, 3–4 mm in height; lamella edge even or entire, sometime undate; lamellulae in 3–4 tiers. Stipe 28.0–41.1 × 1.8–2.7 mm, cylindrical, central, equal with an enlarged base and nearly subclavate, pale (7–8A6) to pastel red (7A4–5, 8A4–5), smooth, basal mycelium white (1A1); stipe context stuffed, pastel red. Odor and taste not observed.

Basidia 29–38 × 9–13 μm, (mean length = 32 ± 2.5, mean width = 11 ± 1.2), clavate, mostly 4-spored, rarely 2-spored, sterigmata 5–8 μm × 2–3 μm, (mean length = 6.0 ± 1.22, mean width = 2.4 ± 0.45). Basidiospores (excluding ornamentation) [150/3/2] (6.0–) 7.1–11.0 (–12.0) × (6.5–) 7.0–10.4 (–10.9) μm, (mean length = 8.9 ± 0.83, mean width = 8.4 ± 0.71), Q = 1.00–1.36, Q_m_ = 1.08 ± 0.07, globose to subglobose, hyaline, echinulate, spines 2–3 μm long, ca. 1–2 μm wide at the base, crowded. Cheilocystidia 20–31 × 6–9 μm, (mean length = 25 ± 3.5, mean width = 7 ± 1.0), narrowly clavate, thin-walled, colorless and hyaline, abundant. Pleurocystidia 15–31 × 6–8 μm, (mean length = 21 ± 4.2, mean width = 7 ± 0.8), narrowly clavate to subclavate, flexuose or mucronate, thin-walled, hyaline, abundant. Lamellar trama 50–70 μm thick, regular, composed of slightly thick-walled, filamentous hyphae 2–8 μm wide. Lamellar edge more in number of sterile basidia. Subhymenium 7–10 μm thick, tightly interwoven, fusiform or irregular cells, 5–8 × 3–4 μm, (mean length = 7 ± 0.8, mean width = 3.6 ± 0.5). Pileipellis 70–100 μm thick, orange hyaline in KOH, composed of appressed, parallel, simply septate, thin-walled, cylindrical, filamentous hyphae 4–6 μm wide, colorless and hyaline. Stipitipellis composed of appressed, parallel, simply septate, thick-walled, hyphae 3–7 μm wide; stipe trama composed of longitudinally arranged, pastel red in KOH, clavate terminal cells, infrequently branching, septate, thick-walled, hyphae hyaline 3–10 μm wide. Caulocystidia not seen. Clamp present at some septa in pileipellis, lamellae and stipitipellis.

##### Habitat and phenology.

Scattered, gregarious, or caespitose on the ground in the *Fagus* and *Dipterocarpus*.

##### Additional specimens examined.

Thailand. Chiang Mai Province: Mae On District, Huay Keaw Sub-district, elevation 799 m. 6 September 2020, S. M. Tang, HKAS110664; ibid., 6 September 2020, S. M. Tang, HKAS110663.

##### Notes.

**Figure 1.**
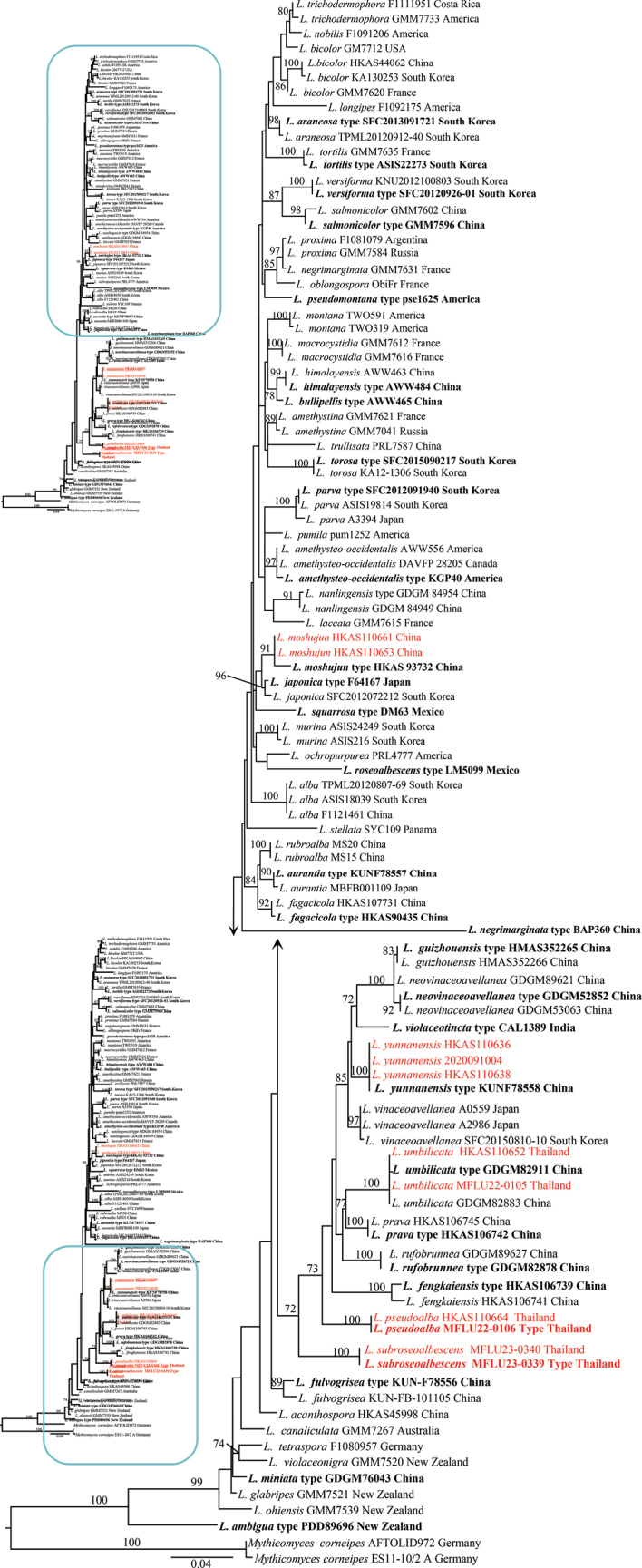
Maximum likelihood tree based on ITS1-5.8S-ITS2. Bootstrap support values ≥ 70%. The new sequences are highlighted in red, and the holotype of each species is in bold.

In our single gene (Fig. 1) phylogenetic analysis, the phylogenetic position of *L.fengkaiensis*, *L.prava*, *L.vinaceoavellanea*, *L.violaceotincta*, *L.umbilicata* and *L.yunnanensis*, within *L.pseudoalba* is well supported (100/1.00) as monophyletic clades. However, *L.yunnanensis* has bigger basidiomata (pileus 60–100 mm wide), brownish to flesh-colored pileus, and relatively bigger basidia (45–50 × 9–10 μm) (Popa et al. 2014). *Laccariavinaceoavellanea* has vinaceous-buff pileus, and rare pileocystidia (Li 2020). *Laccariaviolaceotincta* has dark brown to reddish brown pileus and pleurocystidia absent (Latha and Raj 2019). *Laccariafengkaiensis* has relatively larger basidiomata (pileus 50–90 mm), more obvious striate, stipitipellis hyphal ends are either ascending or aggregating into scattered clusters, smaller basidiospores (5.2–6.3 × 5.1–6.3 μm) and narrower basidia (30–45 × 6–8.5 μm) (Li 2020). *Laccariaprava* has larger basidiomata (pileus 30–75 mm), presence of caulocystidia, and absence of pleurocystidia (Li 2020).

**Figure 2.**
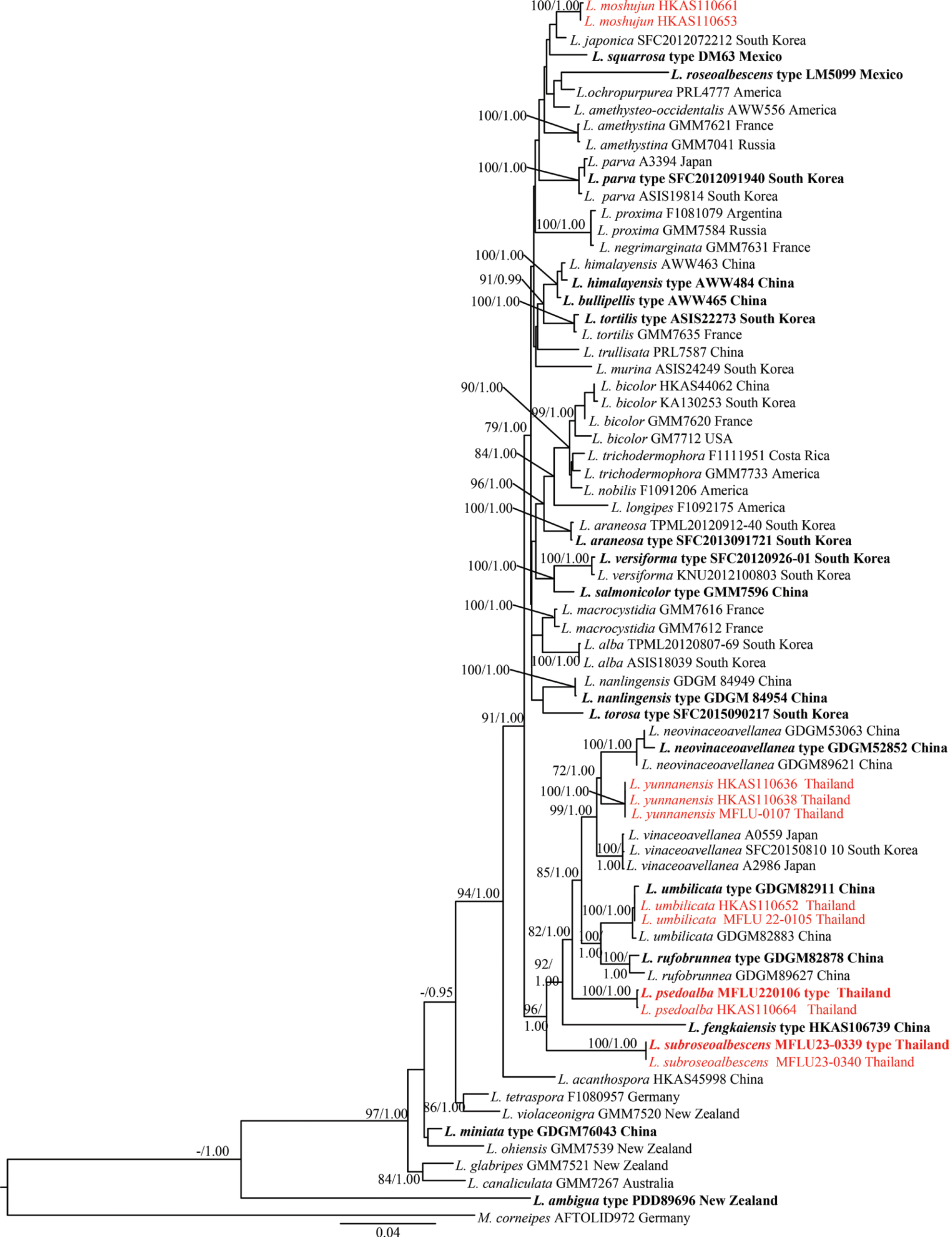
Maximum likelihood phylogeny using ITS1-5.8S-ITS2, LSU, *rpb*2, and *tef*1 sequence data to identify species of *Laccaria* growing on roots of *Mythicomycescorneipes*. ML bootstrap (≥70%) and posterior probabilities (≥0.90) are indicated above branches or in front of the branch leading to each node. The new species and a new record are highlighted in red; the holotype of each species is in bold.

**Figure 3.**
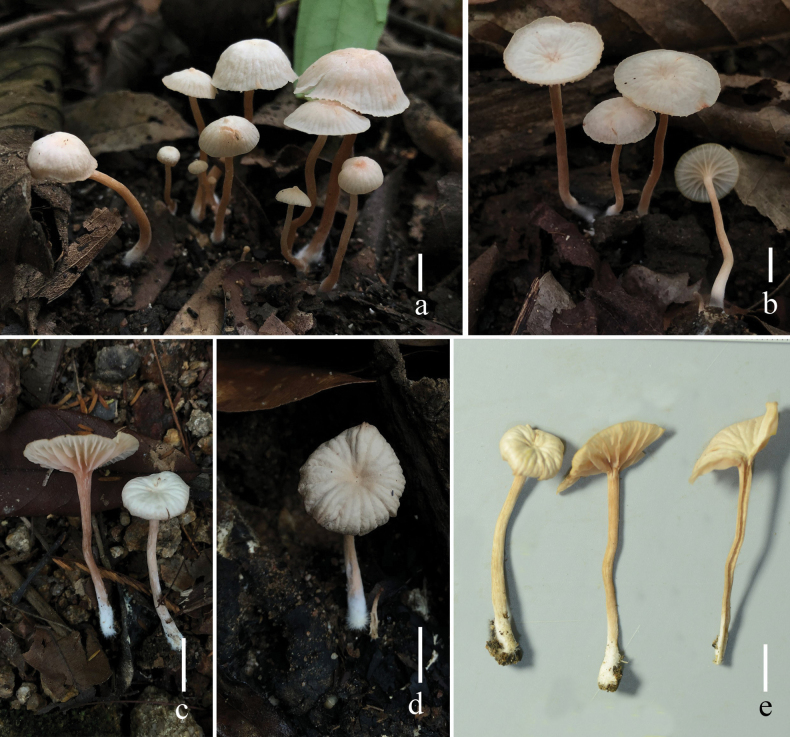
Fresh basidiomata of *Laccariapseudoalba* (**a** holotype, MFLU 22-0106 **b, e** HKAS 110664 **c, d** HKAS 110663). Scale bars: 5 mm. Photographs by Song-Ming Tang.

**Figure 4.**
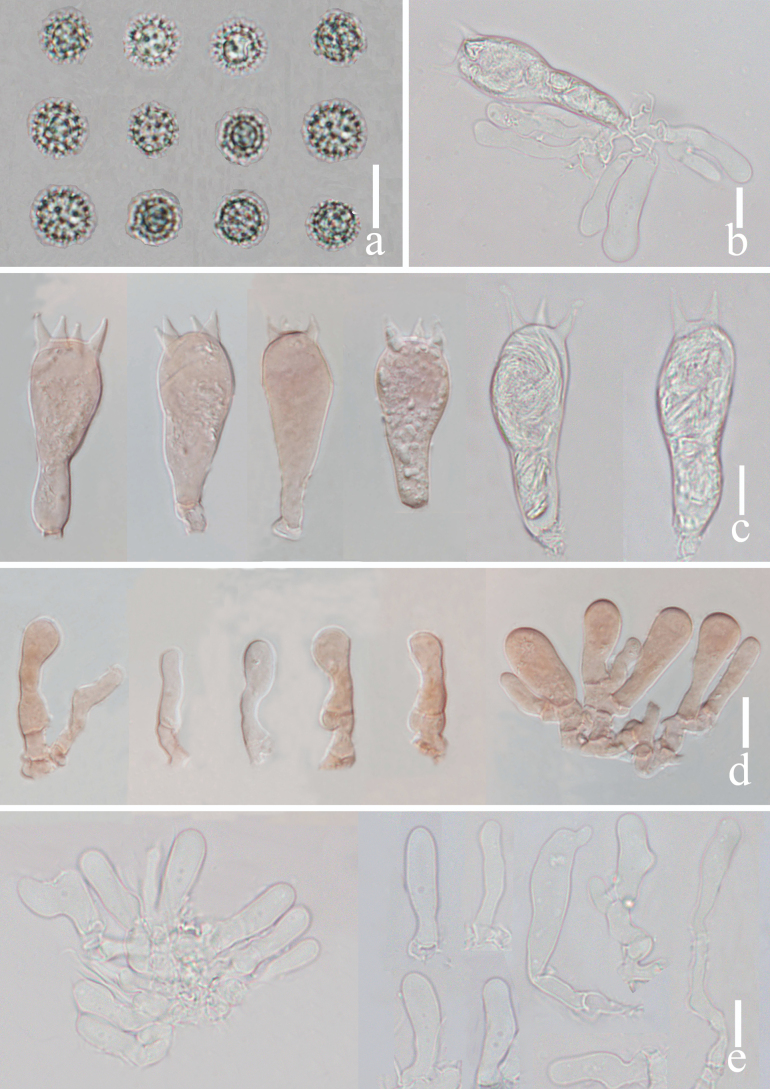
*Laccariapseudoalba***a** basidiospores **b** basidium and basidioles **c** basidia **d** cheilocystidia **e** pleurocystidia. Scale bars: 10 μm. Photographs by Song-Ming Tang.

**Figure 5.**
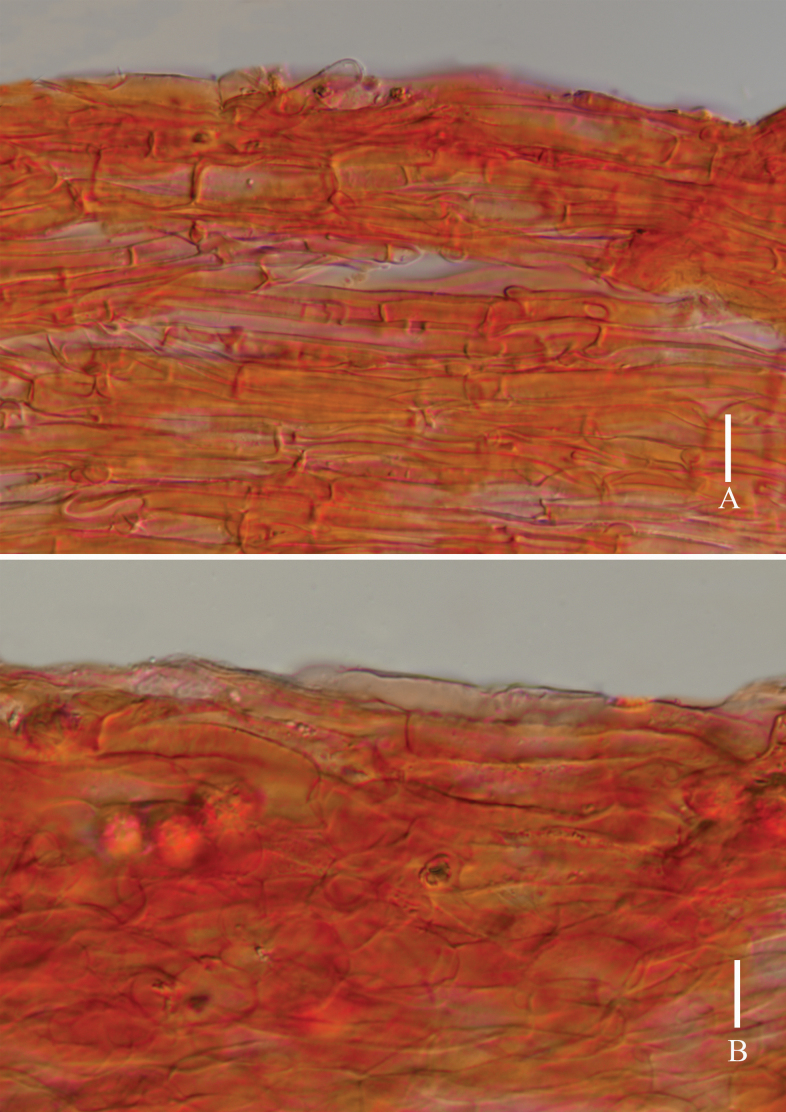
*Laccariapseudoalba***A** stipitipellis **B** pileipellis. Scale bars: 10 μm. Photographs by Song-Ming Tang.

*Laccariapseudoalba* can be confused with *Laccariaalba* Zhu L. Yang & Lan Wang due to their similar orange-white to whitish basidiomata. However, *L.alba* has white to whitish stipe while *L.pseudoalba* has pale to pastel red stipe, relatively thicker pileipellis (30–75 μm), absent pleurocystidia, narrower cheilocystidia (4–6 μm), and present clavate, hyaline caulocystidia (Wang et al. 2004).

#### ﻿ Laccaria subroseoalbescens

Taxon classificationFungiAgaricalesHydnangiaceae

S.M. Tang & S.H. Li sp. nov.

379B9AEB-3B92-52FB-809A-22049472A2F6

853964

Figs 6 , 7 , 8 , 15

##### Etymology.

The epithet “*subroseoalbescens*” refers to its similarity to *L.roseoalbescens* in their pale orange to greyish orange and clearly striate on the pileus surface.

##### Holotype.

Thailand. Chiang Rai Province: Thasud, Muang District, Mae Fah Luang University Park, elevation 488 m, dominated by *Dipterocarpus* sp., 10 August 2020, OR1663 (MFLU23-0339).

Basidiomata small. Pileus 2–8 mm in diam., plano-concave to concave, glabrous, pale yellow (4A3), light yellow (4A4), pale orange (5A3) to greyish orange (5A4), light orange at center, becoming paler towards the margin, without umbo, when loss of moisture or with age becoming whitish, clearly striate on the surface; context thin, below 1 mm, pale orange (5A2–3), unchanging. Lamellae distant, arcuate, adnate with decurrent tooth, pale pink (6–7A2), 1–2 mm in height; lamella edge even or entire, sometime undate; lamellulae in 2–3 tiers. Lamellae pale pink (6–7A2) to bright flesh-pink, 1.2 mm diam., subdecurrent or decurrent, thick, regular, close. Stipe 5.0–13.0 × 0.8–1.7 mm, cylindrical, central or eccentric, equal with an enlarged base and nearly subclavate, pale pink (7A2) to light orange (6A5), concolorous with pileus, becoming whitish after loss of moisture or with age, smooth; stipe context stuffed, pale orange. Odor and taste not observed.

**Figure 6.**
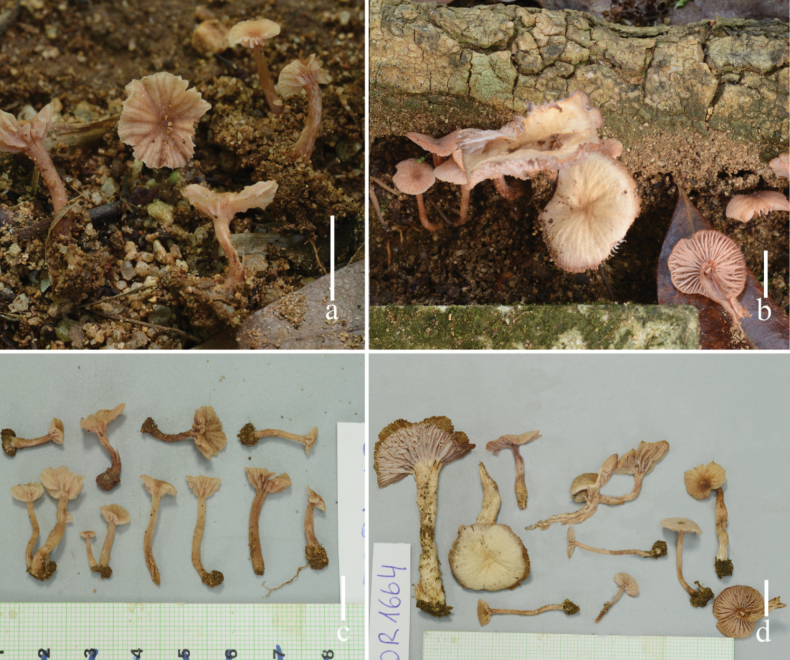
Fresh basidiomata of *Laccariasubroseoalbescens* (holotype **a, c** OR 1663, MFLU23-0339 **b, d** OR 1664, MFLU23-0340). Scale bars: 1 cm.

Basidia 30–46 × 8–14 μm, av. 38 ± 4.1 × 13.8 ± 1.3 μm, clavate, mostly 4–spored, rarely 2–spored; sterigmata 6–14 μm × 2–4 μm, av. 8.5 ± 2.9 × 3.3 ± 0.8 μm. Basidiospores [78/2/2] 7.0–8.9 × 6.8–9.0 μm, av. 8.3 ± 0.6 × 7.8 ± 0.6 μm, Q_m_ = 1–1.3, Q_av_. = 1.1 ± 0.08, globose, hyaline; echinulate spines 2–3 × 1–2 μm, crowded. Cheilocystidia 23–37 × 4–8 μm, av. 34 ± 8.5 × 6.5 ± 1.5 μm, narrowly clavate, thin-walled, colorless and hyaline, abundant. Pleurocystidia 36–59 × 5–8 μm, av. 48 ± 7.6 × 6.5 ± 1.3 μm, subclavate, narrowly clavate, flexuose or mucronate, thin-walled, hyaline hyphae. Lamellar edge more in number of sterile basidia, composed of clavate, cylindrical inflated cells 11–23 × 8–15 μm, thin-walled, colorless, similar to basidioles in shape. Subhymenium 10–24 μm thick, tightly interwoven, fusiform or irregular cells, 4–7 × 5–6 μm. Lamellar trama 74–90 μm thick, regular, composed of slightly thick-walled, filamentous hyphae 2–5 μm wide. Pileipellis 60–90 μm thick, colorless hyaline in KOH, composed of appressed, parallel, simply septate, thin-walled, cylindrical, filamentous hyphae 7–11 μm wide, colorless and hyaline. Stipitipellis composed of appressed, parallel, simply septate, thick-walled, hyphae 3–5 μm wide; stipe trama composed of longitudinally arranged, pastel red in KOH, clavate terminal cells, infrequently branching, septate. Caulocystidia abundant, flexuose, thin-walled, hyaline hyphae, 4–5 μm wide. Clamp present at some septa in pileipellis, lamellae and stipitipellis.

**Figure 7.**
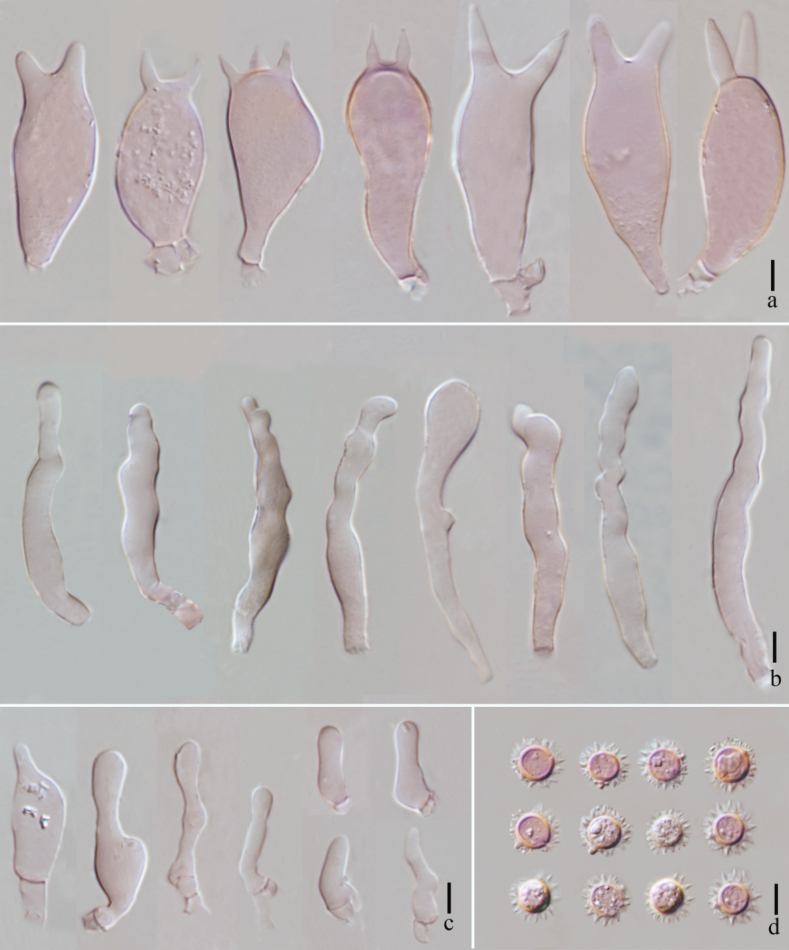
*Laccariasubroseoalbescens* (OR1663, MFLU23-0339) **a** basidia **b** cheilocystidia **c** pleurocystidia **d** basidiospores. Scale bars: 10 μm.

##### Habitat and phenology.

Scattered on the ground in subtropical forests of *Dipterocarpus*.

**Figure 8.**
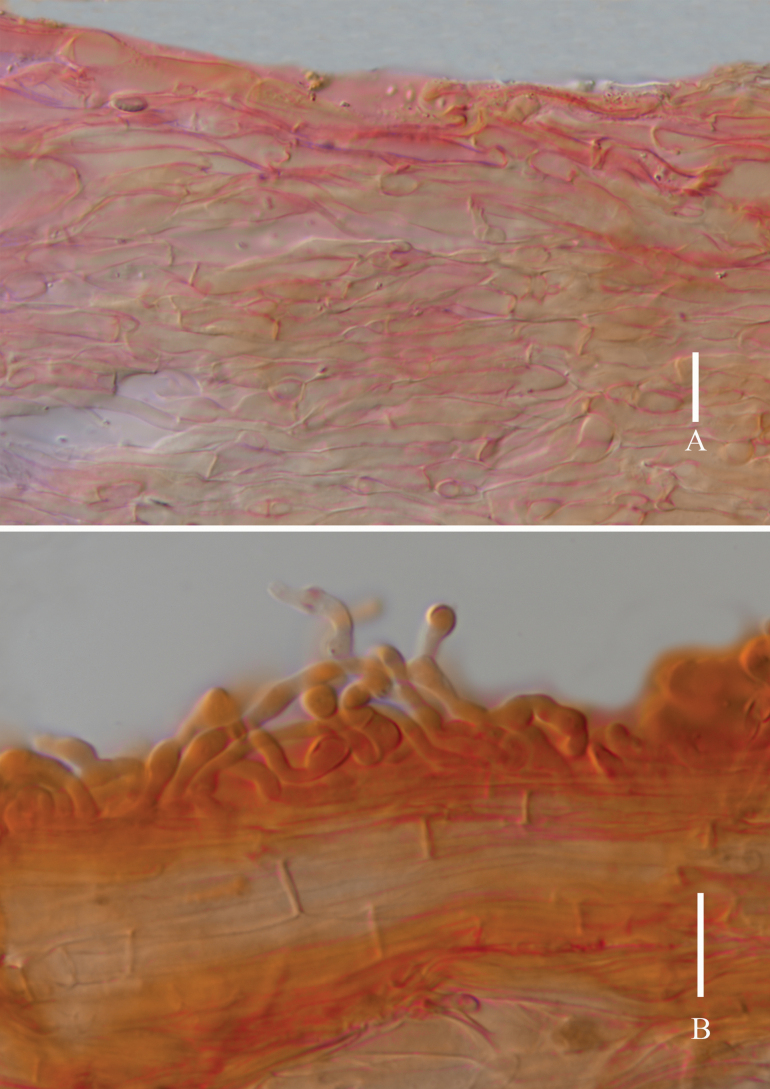
*Laccariasubroseoalbescens* (OR1663, MFLU23-0339) **A** pileipellis **B** stipitipellis. Scale bars: 10 μm. Photographs by Song-Ming Tang.

##### Additional specimens examined.

Thailand. Chiang Rai Province: Thasud, Muang District, Mae Fah Luang University, 10 August 2020, elev. 489 m, OR1664 (MFLU23-0340).

##### Notes.

In single gene (Fig. 1) phylogenetic analysis, *L.subroseoalbescens* is closely related to *L.pseudoalba.* However, *L.pseudoalba* has a pale orange to orange white pileus, and larger basidiospores 7.1–11.0 × 7.0–10.4 μm, shorter basidia sterigmata (5–8 μm × 2–3 μm).

*Laccariaacanthospora* A.W. Wilson & G.M. Muell., *L.ambigua* K. Hosaka, A.W. Wilson & G.M. Mueller, and *L.negrimarginata* A.W. Wilson & G.M. Mueller have similar small basidiomata (pileus ≤ 15 mm) as *L.subroseoalbescens*. However, *L.acanthospora* has orange pileus, relatively longer spines (2–6 μm) on the basidiospores, and longer basidia (40–56 × 10–14 μm) (Wilson et al. 2013). *Laccariaambigua* has orange-brown basidiomata, without the striates on the pileus margin, and stipe orange-brown to ochraceous buff (Wilson et al. 2017). *Laccarianegrimarginata* has dark blackish brown to dark brown pileus and stipe, fibrillose to appressed squamulose on the pileus surface (Wilson et al. 2013).

*Laccariaindohimalayana* K. Das, I. Bera & Vizzini and *L.roseoalbescens* T.J. Baroni, Montoya & Bandala are similar to *L.subroseoalbescens* in their sharing a light yellow basidiomata. However, *L.indohimalayana* doesn’t have cheilocystidia and pleurocystidia, and is clearly separated in the phylogeny (Wang et al. 2019). *Laccariaroseoalbescens* has larger pileus (7–29 mm), and shorter echinae (1–2.5 μm) (Montoya et al. 2015).

#### ﻿ Laccaria umbilicata

Taxon classificationFungiAgaricalesHydnangiaceae

Ming Zhang, in Zhang, Gao, Mu & Deng, Journal of Fungi 9(12, no. 1179): 16 (2023)

231BBE65-13BD-51E6-999C-138DC5B72702

**Figure 9.**
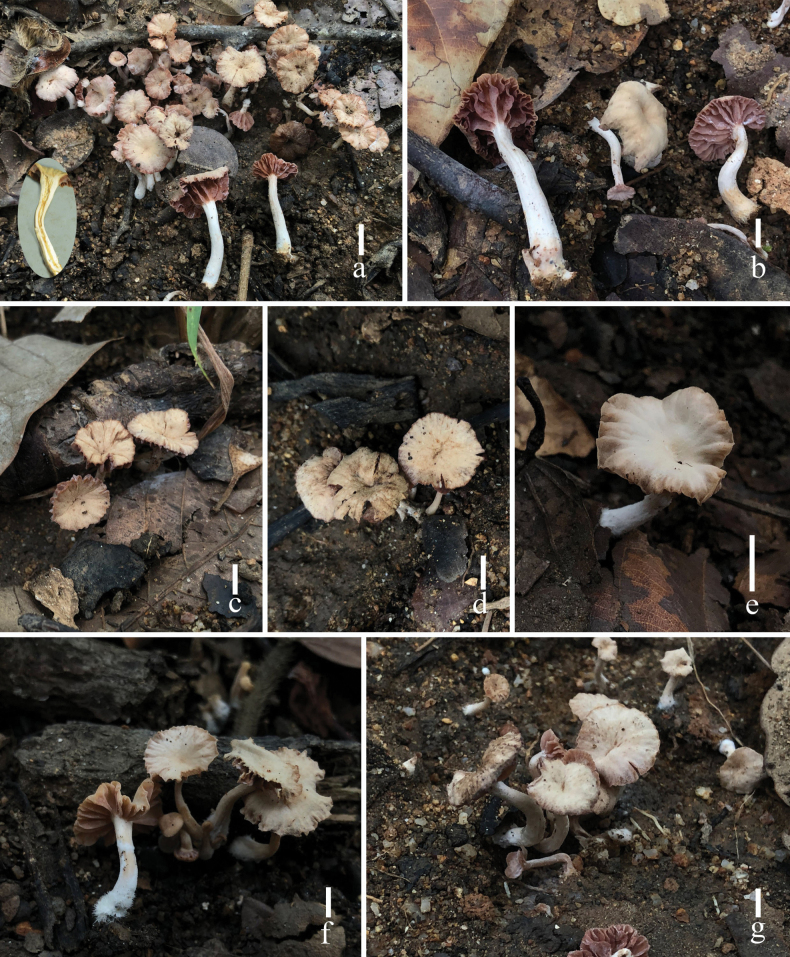
Fresh basidiomata of *Laccariaumbilicata* (**a–d**MFLU 22-0105, **e, f** HKAS 110652, **g** HKAS 110651). Scale bars: 5 mm. Photographs by Song-Ming Tang.

**Figure 10.**
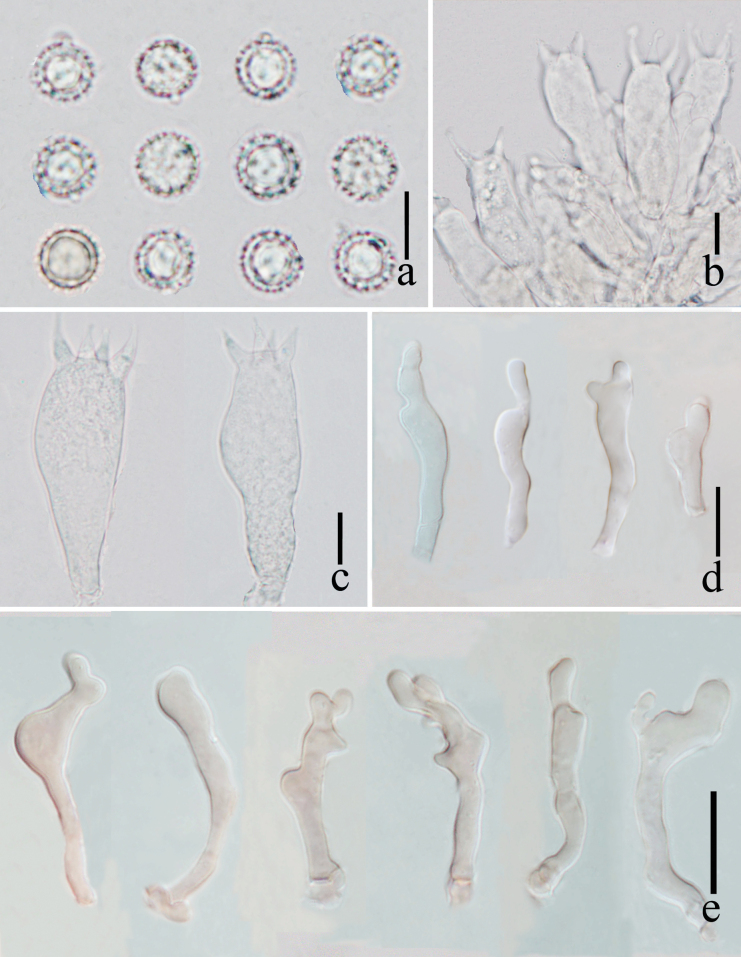
*Laccariaumbilicata* (MFLU 22-0105) **a** basidiospores **b–c** basidia **d** cheilocystidia **e** pleurocystidia. Scale bars: 10 μm. Photographs by Song-Ming Tang.

**Figure 11.**
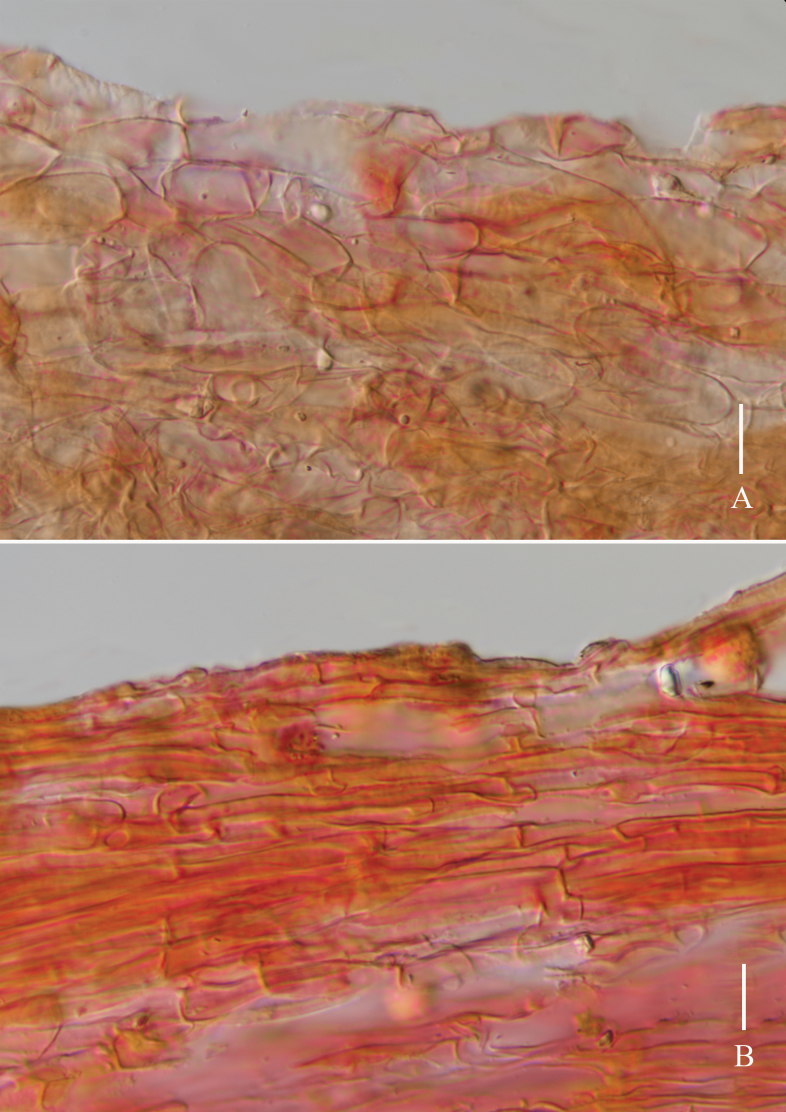
*Laccariaumbilicata***A** pileipellis **B** stipitipellis. Scale bars: 10 μm. Photographs by Song-Ming Tang.

Figs 9 , 10 , 11 , 15

##### Description.

Basidiomata small. Pileus 7–11 mm in diam., applanate to plano-concave, depressed to subumbilicate shape of center, light yellow (1B4), when loss of moisture or with age becoming whitish, clearly striate towards the margin on the surface, without umbo; margin straight, eroded of margin; context thin, 0.5–1 mm, light yellow (1B4). Lamellae reddish brown (8E5–8), 3–5 mm wide; lamellulae subdecurrent to decurrent, thick, regular, distant, 3–4 mm in height; lamella edge even or entire, sometime undate, lamellulae in 3–4 tiers. Stipe 11.0–18.0 × 1.4–2.0 mm, cylindrical, fistulose, central or eccentric, equal with an enlarged base and nearly subclavate, white (1A1), sometime pale orange, basal mycelium white (1A1); stipe context fistulose, white, sometime pale orange. Odor and taste not observed.

Basidia 30–49 × 9–15 μm, (mean length = 39 ± 6.3, mean width = 12 ± 1.9), clavate, hyaline, 4-spored; sterigmata 5–8 × 2–3 μm (mean length = 6 ± 0.7, mean width = 2.5 ± 0.23). Basidiospores (excluding ornamentation) [150/3/2] (6.4–) 7.9–11.0 (–12.0) × (5.7–) 7.4–9.6 (–10.8) μm, (mean length = 9.4 ± 0.76, mean width = 8.9 ± 0.73), Q = 1.00–1.34, Q_m_ = 1.07 ± 0.06, globose to subglobose, hyaline, echinulate; spines 0.2–0.5 μm long, ca. 0.5–0.8 μm wide at the base, crowded. Cheilocystidia 15–20 × 3–5 μm, (mean length = 17 ± 1.7, mean width = 4 ± 0.8), subclavate, narrowly clavate, hyphae-like, flexuose or mucronate, thin-walled, hyaline, abundant. Pleurocystidia 17–25 × 4–6 μm, (mean length = 20 ± 2.6, mean width = 5 ± 0.7), subclavate, narrowly clavate, hyphae-like, flexuose or mucronate, thin-walled, hyaline, abundant. Lamellar trama regular, 50–70 μm wide, composed of slightly thick-walled, filamentous hyphae 2–8 μm wide. Subhymenium 7–11 μm thick, tightly interwoven, fusiform or irregular cells, 5–10 × 2–3 μm, (mean length = 8 ± 1.2, mean width = 2.3 ± 0.3). Lamellar edge heteromorphous, more in number of cheilocystidia. Pileipellis 60–100 μm thick, composed of interwoven radiating, thin-walled, cylindrical, filamentous hyphae 3–8 μm wide. Stipitipellis composed of appressed, parallel, simply septate, thick-walled, hyphae 2–8 μm wide; stipe trama composed of longitudinally arranged, pale orange in KOH, clavate terminal cells, infrequently branching, septate, thick-walled, hyphae 8–20 μm wide. Caulocystidia not seen. Clamp present at some septa in pileipellis, lamellae and stipitipellis.

##### Habitat and phenology.

Gregarious or caespitose on the ground associated with the *Fagus* and *Dipterocarpus*.

##### Additional specimens examined.

Thailand, Chiang Mai Province: Mae On District, Huay Keaw, Pox Village, elevation 795 m., 6 September 2020, S. M. Tang, HKAS110652; ibid., 6 September 2020, S. M. Tang, HKAS110651. Chiang Mai Province, Mae On District, Huay Keaw, elevation 812 m, 6 September 2020, S. M. Tang, 2020090626 (MFLU 22-0105)

##### Notes.

Following BLASTn searches of NCBI GenBank, the closest matches of the ITS and LSU sequences of our new collection (HKAS110652) is *L.umbilicata* (specimen GDGM82911 (holotype) ITS 99.67% shared identity; specimen GDGM82883 LUS 99.54% shared identity). The morphology of Thai collections was fit to the original description of *L.umbilicata* from Southwest China (Yunnan) by Zhang et al. (2023), including small basidiomata (10–28 mm), pale yellow, pale orange to light orange pileus, and clavate to ellipsoid pleurocystidia. Phylogenetically, our specimens grouped with *L.umbilicata* GDGM82911 (holotype) have high support values (Fig. 1, 100). Thus, we identified this specimen as a new record from Thailand.

#### ﻿ Laccaria yunnanensis

Taxon classificationFungiAgaricalesHydnangiaceae

F. Popa, Rexer, G. Kost, Mycol. Progress 13(4): 1113 (2014)

B108C03A-7955-5696-BD4E-20E5125952F2

**Figure 12.**
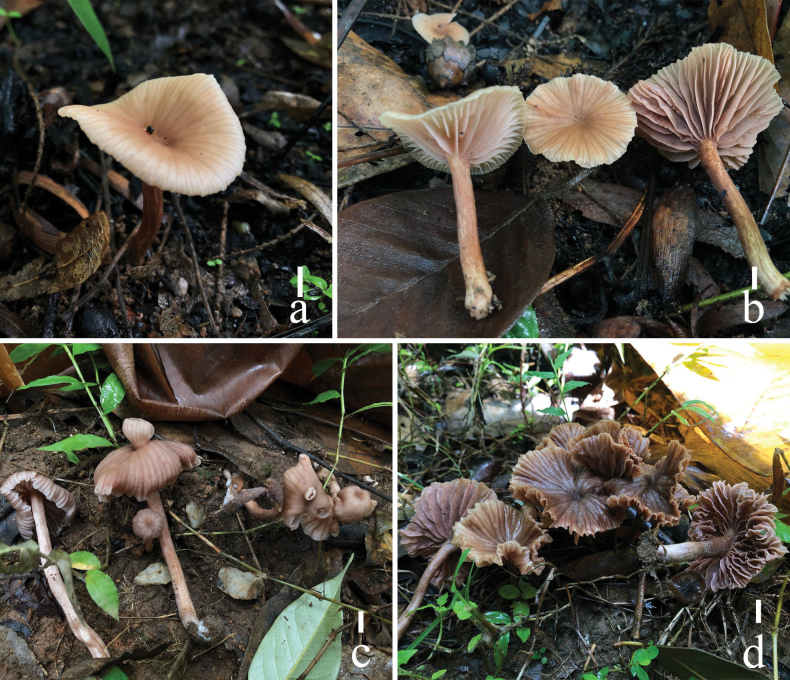
*Laccariayunnanensis***a–d** basidiomata (**a, b** HKAS 110638 **c** HKAS 110631 **d** HKAS 110630). Scale bars: 1 cm. Photographs by Song-Ming Tang.

**Figure 13.**
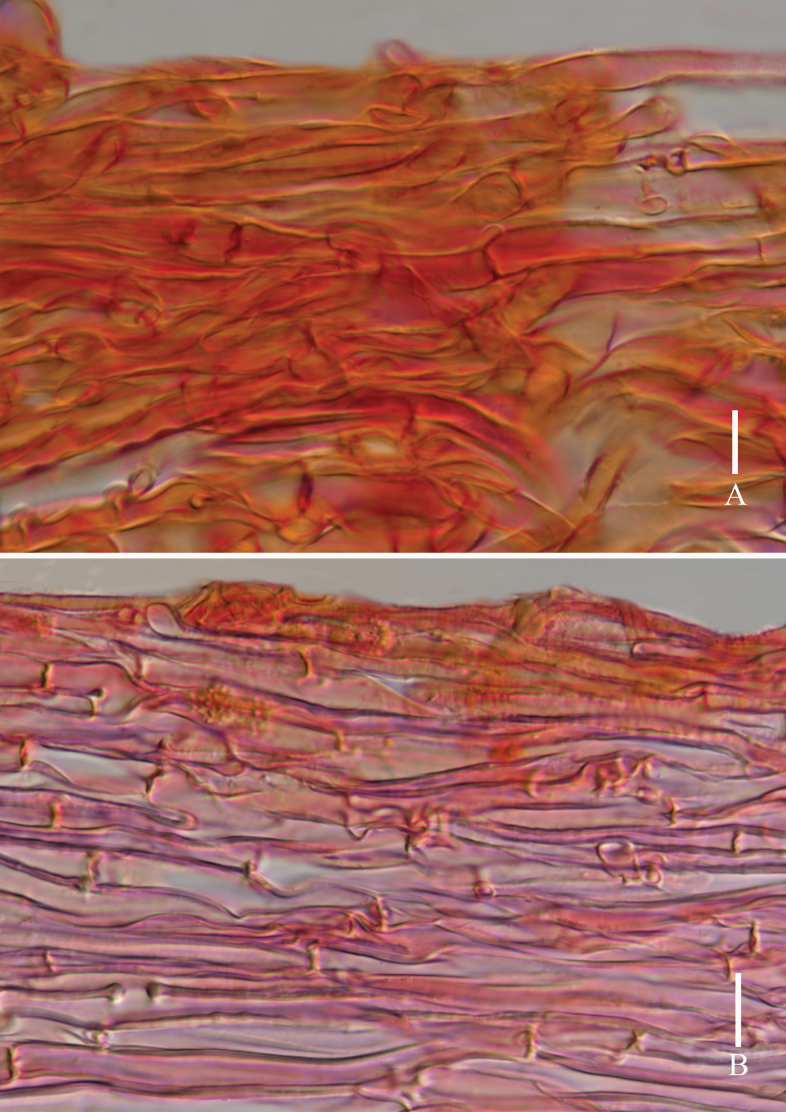
*Laccariayunnanensis***A** pileipellis **B** stipitipellis. Scale bars: 20 μm. Photographs by Song-Ming Tang.

**Figure 14.**
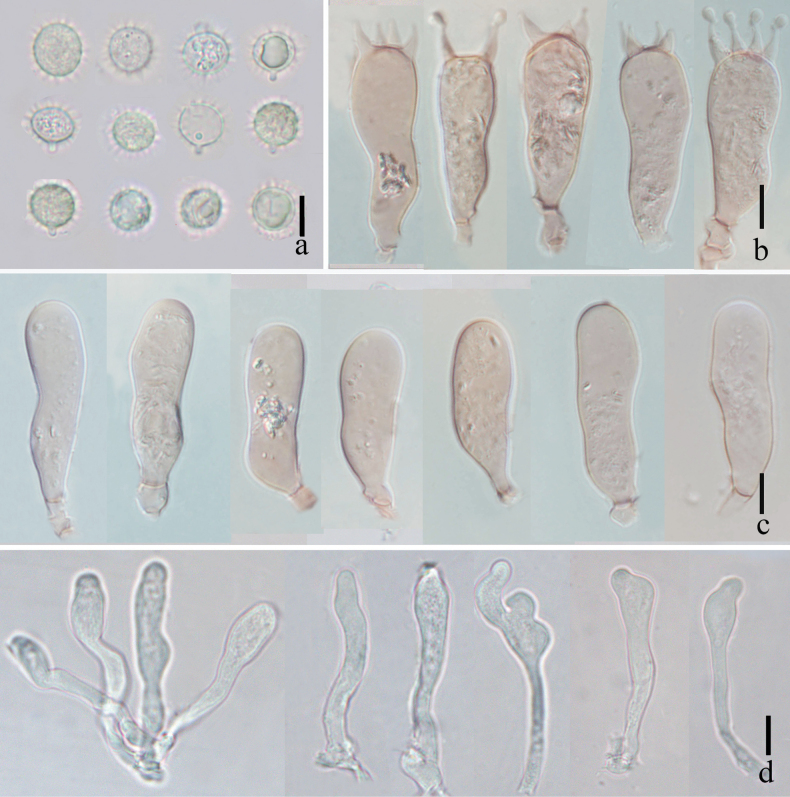
*Laccariayunnanensis***a** basidiospores **b** basidia **c** cheilocystidia **d** pleurocystidia. Scale bars: 10 μm. Photographs by Song-Ming Tang.

**Figure 15.**
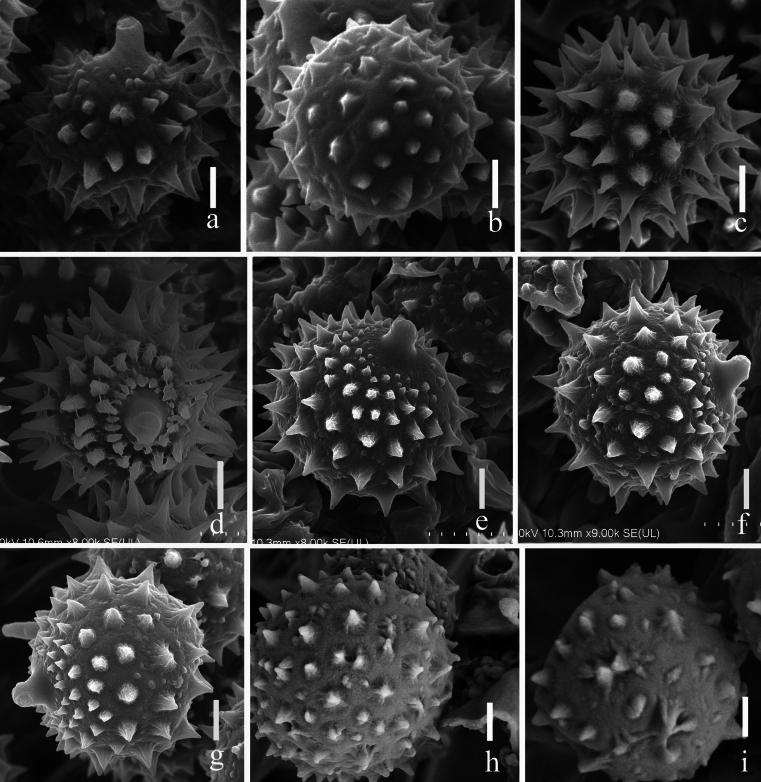
Characteristics of basidiospores ornamentations **a, b***Laccariapseudoalba***c, d***Laccariasubroseoalbescens***e–g***Laccariaumbilicata***h–i***Laccariayunnanensis.* Scale bars: 2 μm. Photographs by Song-Ming Tang.

Figs 12 , 13 , 14 , 15

##### Description.

Basidiomata large. Pileus 50–110 mm in diam., plano-concave, concave to hemisphaericus, glabrous, without umbo, yellowish-brown (5D4–5D8), brown (6E5–6E8), dark brown (7–8F5–8), yellowish brown when young, becoming dark brown with age, clearly striate on the surface; margin inflexed, sometimes reflexed; context thin, 2–3 mm, yellowish brown (5D4). Lamellae adnate, distant, yellowish-brown (5D4–5D8), brown (6E5–6E8), dark brown (7–8F5–8), 5–8 mm in height; lamella edge even or entire, sometime undate; lamellulae subdecurrent or decurrent, thick, regular, close; lamellulae in 2–3 tiers. Stipe 24.2–77.8 × 2.1–5.6 mm, cylindrical, central or eccentric, equal, smooth, same color as the pileus, yellowish-brown (5D4–8), brown (6E5–8), dark brown (7F5–8, 8F5–8), to whitish at the base, basal mycelium white (1A1); stipe context stuffed, yellowish brown. Odor and taste not observed.

Basidia 41–53 × 7–15 μm, (mean length = 45 ± 8.3, mean width = 10 ± 2.3), clavate, mostly 4–spored, rarely 2–spored, sterigmata 6–9 μm, 2–3 μm wide at base. Basidiospores (excluding ornamentation) [250/5/2] 7.9–10.9 × 8.0–10.9 μm, (mean length = 9.5 ± 0.81, mean width = 9.4 ± 0.73), Q = 1.00–1.21, Q_m_ = 1.12, globose, hyaline, echinulate, spines 1–2 μm long, ca. 0.5–1.0 μm wide at the base, crowded. Pleurocystidia 50–70 × 10–25 μm, (mean length = 59 ± 5.4, mean width = 18 ± 2.4), clavate to ellipsoid, thin-walled, hyaline hyphae. Cheilocystidia abundant, 25–50 × 4–8 μm, (mean length = 38 ± 3.5, mean width = 6 ± 0.8), subclavate, narrowly clavate to cylindrical, flexuose or mucronate, thin-walled, hyaline hyphae. Lamellar trama 60–100 mm thick regular, composed of slightly thick-walled, filamentous hyphae 2–12 μm wide. Lamellar edge more in number of cheilocystidia. Subhymenium 7–10 μm thick, tightly interwoven, fusiform or irregular cells, 2–4 × 4–5 μm, (mean length = 3 ± 0.2, mean width = 4.3 ± 0.3). Pileipellis 40–80 μm thick, yellowish brown in KOH, composed of radiating interwoven, thin-walled, cylindrical, filamentous hyphae 3–5 μm wide. Stipitipellis composed of appressed, parallel, simply septate, thick-walled, hyphae 5–20 μm wide; stipe trama composed of longitudinally arranged, pale yellowish in KOH, clavate terminal cells, infrequently branching, septate, thick-walled, hyphae 6–18 μm wide. Caulocystidia not seen. Clamp present at some septa in pileipellis, lamellae and stipitipellis.

##### Habitat and phenology.

Scattered, gregarious, or caespitose on the ground in *Dipterocarpus* and *Fagus*.

##### Material examined.

Thailand. Chiang Mai Province: Mare Taeng District, Pha Deng Village, 14 July 2020, S. M. Tang, HKAS 110638; ibid., 14 July 2020 S. M. Tang, HKAS 110636; ibid., 11 August 2020 S. M. Tang, MFLU 22-0107; ibid., 10 September 2020 F. M. Yu, HKAS 110630.

##### Notes.

The morphology of Thai collections fit the original description of *L.yunnanensis* from Southwest China (Yunnan) by Popa et al. (2014) including large basidiomata (pileus 50–110 mm in diam.), yellowish brown, brown, dark brown, yellowish brown or dark brown pileus, basidia clavate, and clavate to ellipsoid pleurocystidia. Our molecular analysis also indicated that four Thai collections belong to the same species.

## ﻿Discussion

With the development of molecular phylogenetic analysis, many new *Laccaria* species have been rapidly described (Wilson et al. 2013, 2017; Popa et al. 2014, 2016; Luo et al. 2016; Cho et al. 2018; Li 2020; Cui et al. 2021; Zhang et al. 2023). Morphological characteristics and systematically informative traits are few in *Laccaria*; hence, molecular analyses are important for classification and species identification. In this study, we used the molecular phylogenetic analysis (single gene ITS1+ITS2 and 5.8 S Fig. 1), and multi-locus phylogenetic analysis (ITS, LSU, *RPB2* and *TEF1* sequences Fig. 2) to evaluate the taxonomy of *Laccaria* in Thailand. We identified 2 new species, namely *L.pseudoalba*, *L.subroseoalbescens*, and two new records, *L.umbilicata* and *L.yunnanensis*.

Before this study, five *Laccaria* species, namely *L.amethystina* Cooke, *L.laccata*, *L.ohiensis* (Mont.) Singer, *L.proxima* (Boud.) Pat., and *L.vinaceoavellanea* Hongo were reported to occur in Thailand based on morphological characteristics, but the specimens lacked detailed descriptions (Chandrasrikul et al. 2011). In the future, more extensive specimen collection is needed in Thailand to determine whether these species are indeed distributed there.

So far, only *Fagus* and *Dipterocarpus* have been found to host *L.pseudoalba*, *L.umbilicata* and *L.subroseoalbescens.* Species in *Laccaria* are similar in morphology characters, so habitat and host trees can provide important information for species identification. It is clear that several *Laccaria* species have a wide range of host trees while other species of *Laccaria* associate with a limited group or single host (Mueller 1992). For example, *L.laccata* (Scop.) Cooke (hosts: *Castanea*, *Quercus*, *Pinus*) and *L.himalayensis* A.W. Wilson & G.M. Muell. (hosts: *Abies*, *Pinus*, *Picea*) have been reported with a variety of hosts in forests; whereas *L.trichodermophora* G.M. Muell. (host: *Quercus*) and *L.masoniae* G. Stev. (host: Nothofagus) have only been found with a single host tree species (Mueller 1984) .

To date, 42 species of *Laccaria* have been reported in Asia (Wilson et al. 2013, 2017; Popa et al. 2014, 2016; Luo et al. 2016; Cho et al. 2018; Li 2020; Zhang et al. 2023). These species are described in China (26 species), South Korea (12 species), Japan (seven species), India (seven species), and Thailand (four species, this study). The taxonomy of *Laccaria* species in Thailand is still poorly understood and unclear. As a result of their very similar morphological characteristics, many *Laccaria* species are misidentified as the same species. Thus, for a better understanding of the species diversity of *Laccaria* in Thailand and their relationships within the genus, additional studies and data are required.

## Supplementary Material

XML Treatment for Laccaria pseudoalba

XML Treatment for Laccaria subroseoalbescens

XML Treatment for Laccaria umbilicata

XML Treatment for Laccaria yunnanensis

## ﻿Additional information

### Author ORCIDs

Song-Ming Tang https://orcid.org/0000-0002-6174-7314

Santhiti Vadthanarat https://orcid.org/0000-0002-9035-0375

Bhavesh Raghoonundon https://orcid.org/0000-0001-6671-2404

Zong-Long Luo https://orcid.org/0000-0001-7307-4885

Feng-Ming Yu https://orcid.org/0000-0001-9133-8645

Jun He https://orcid.org/0000-0001-7027-7206

### Data availability

All of the data that support the findings of this study are available in the main text.
